# Development of a Portable Cell-Based Biosensor for the Ultra-Rapid Screening for Boscalid Residues in Lettuce

**DOI:** 10.3390/bios14060311

**Published:** 2024-06-18

**Authors:** Georgia Moschopoulou, Vasileios Tsekouras, Josep V. Mercader, Antonio Abad-Fuentes, Spyridon Kintzios

**Affiliations:** 1 Laboratory of Cell Technology, Department of Biotechnology, Agricultural University of Athens, European University for Smart Urban Coastal Sustainability, Iera Odos 75, 11855 Athens, Greece; tsekouras@aua.gr (V.T.); skin@aua.gr (S.K.); 2 Department Preservation and Food Safety Technologies, Institute of Agrochemistry and Food Technology (IATA-CSIC), Av. Agustí Escardino 7, 46980 Paterna, Spain; jvmercader@iata.csic.es (J.V.M.); aabad@iata.csic.es (A.A.-F.)

**Keywords:** Bioelectric Recognition Assay (BERA), biosensor, boscalid, cell-based biosensor, Molecular Identification through Membrane Engineering (MIME), pesticide residue

## Abstract

Fungal plant pathogens have posed a significant threat to crop production. However, the large-scale application of pesticides is associated with possible risks for human health and the environment. Boscalid is a widely used fungicide, consistently implemented for the management of significant plant pathogens. Conventionally, the detection and determination of boscalid residues is based on chromatographic separations. In the present study, a Bioelectric Recognition Assay (BERA)-based experimental approach combined with MIME technology was used, where changes in the electric properties of the membrane-engineering cells with anti-boscalid antibodies were recorded in response to the presence of boscalid at different concentrations based on the maximum residue level (MRL) for lettuce. The membrane-engineering Vero cells with 0.5 μg/mL of antibody in their surface were selected as the best cell line in combination with the lowest antibody concentration. Furthermore, the biosensor was tested against another fungicide in order to prove its selectivity. Finally, the BERA cell-based biosensor was able to detect the boscalid residue, below and above the MRL, in spiked lettuce leaf extracts in an entirely distinct and reproducible manner. This study indicates that the BERA-based biosensor, after further development and optimization, could be used for the routine, high-throughput detection of boscalid residue in lettuce, and not only that.

## 1. Introduction

Fungal plant pathogens constitute a significant threat to crop production since the emergence of agriculture, causing extensive damage and losses through their infections. In response, a variety of chemical compounds has been extensively utilized, to mitigate or prevent the negative effects of phytopathogenic fungi and secure food production [1,2]. Nowadays, fungicides and bactericides account for 41% of the total use of pesticides [3] in the European Union (EU); thus, credible predictions are indicating that their application will be increased during the next years [4]. However, the large-scale application of pesticides is associated with possible risks to human health and the environment [5]. To address that, most countries (i.e., in the EU, and the USA) have established strict regulatory frameworks to evaluate threats and ensure the sustainable use of pesticides, while the FAO and the WHO have founded a joint body since 1963, the “Joint Meeting on Pesticide Residues” (JMPR), for harmonizing health-based guidance for pesticide use and maximum residue level (MRL) values [6,7].

One of the most rapidly expanding groups of chemical fungicides is succinate-dehydrogenase inhibitors (SDHIs) [8]. These are broad-spectrum fungicides that act on Krebs cycle enzyme succinate dehydrogenase, inhibiting the mitochondrial respiratory chain complex II [9]. Boscalid is the most prominent member of SDHI agents [10], consistently implemented for the management of significant plant pathogens, including the soilborne fungi *Sclerotinia minor* and *S. sclerotiorum*, *Botrytis cinerea*, *Colletotrichum destructivum*, *Erysiphe necator*, and various *Alternaria* species [11,12,13,14,15]. Despite its effectiveness in managing fungal infections, the extensive usage of boscalid brings up concerns regarding the residues’ fate in soil, crops, water resources, and organisms that are not targeted. Boscalid demonstrates minimal toxicity towards mammals, with an acceptable daily intake (ADI) of 0.04 mg/kg·bw, but it is not considered harmless for humans [10,16]. The fungicide is frequently detected in food and environmental samples by national authorities. A survey on pesticide residues in commercial products in China found 139 pesticides in nine agricultural products, with boscalid being the fourth most abundant. Similarly, a study on bottled wines in Slovenia identified boscalid in 76% of the samples, making it the most frequently detected pesticide. Although extensively used in agriculture, boscalid residues in food samples are typically found at concentrations within the legal limit (0.01–50 ppm). For instance, in the European Union, only 0.1% of samples exceeded maximum residue levels (2–50 ppm) during the 2020 inspections. Nevertheless, several reports highlight potential side effects in non-target species, as prolonged exposure to the fungicide can lead to chronic toxicity in honeybees [17], metabolic disorders in Colorado potato beetle (*Leptinotarsa decemlineata*) [18], and toxicity in aquatic species such as zebrafish, *Daphnia magna*, and *Chlorella vulgaris* [10,19,20]. Due to its high-water solubility, boscalid is also the most abundant pesticide in surface water and groundwater, often exceeding the permissible limit of 0.1 μg/L. 

Pesticide applications are potentially hazardous to humans, non-target organisms, and the environment; therefore, supervising their residues is essential in order to identify and assess potential risks [21,22]. Pesticide monitoring is a multidisciplinary approach, integrating diverse analytical techniques for the effective detection and quantification of residues in environmental matrices. Conventionally, the detection and determination of boscalid residues is based on chromatographic separations by gas or liquid chromatography, frequently coupled with mass spectrometry [23,24,25]. These methods offer a high sensitivity and selectivity, even at trace levels. Another approach implements fluorescent spectroscopy, a technique that is advantageous for the determination of boscalid residues in a non-destructive, rapid, and sensitive procedure [26]. Immunochemical methods, such as the competitive enzyme-linked immunosorbent assay (cELISA) [27] and the monoclonal antibody-based immunoassay [28], have been reported as rapid, sensitive, and cost-effective methods for boscalid monitoring in various crops. Recently, sensor fabrication has emerged as a novel, challenging strategy for the monitoring of pesticide contamination. These devices display significant advantages over traditional methods, including simplicity, sensitivity, selectivity, and the capacity to be deployed in the field [29,30]. An immunosensor, based on surface plasmon resonance, exhibited high recovery rates of boscalid in spiked horticultural crops demonstrating a detection range of 4.5–50 ng/mL [31], whereas a β-cyclodextrin-based sensor presented a high sensitivity, and a detection capacity standing at 2.4 × 10^−6^ ng/mL, significantly lower than the MRL level [32]. Both sensor systems detected boscalid concentrations within a few minutes.

The present study introduces a novel point-of-test system for boscalid detection in lettuce. The novel biosensor is based on Bioelectric Recognition Assay (BERA) and Molecular Identification through Membrane Engineering (MIME) technology. Biosensors typically consist of a biological recognition element that selectively interacts with the analyte, the molecule of interest, and a transducer that converts the biological response into a measurable signal [33]. The Bioelectrical Recognition Assay (BERA) employs mammalian cells immobilized in a gel matrix as recognition elements of specific ligands, which either bind to the cells or affect their physiology. The cell–ligand reactions produce measurable electrophysiological responses [34], since the dynamic changes of the cell membrane potential generate electrical signals [35,36]. The cells’ ability for the specific recognition of the analyte reflects the sensitivity of a BERA biosensing system. Consequently, the identification of a target molecule with cellular biorecognition elements can be upscaled by embedding target-specific receptor molecules onto the surface of the cells. MIME technology is a biotechnological approach that enhances the cell membrane’s selectivity against target molecules, through the electroinsertion of specific antibodies or enzymes on the cell surface [37,38]. BERA has been applied before for the detection of insecticide residues [39,40], while biosensors based on BERA and MIME technology have been constructed for the identification of toxins such as 2,4,6-trichloroanisole [41], viruses [42], reactive oxygen species [43], and antigens [44]. In this case, the proposed biosensor successfully identified the boscalid residue level in spiked lettuce samples.

## 2. Materials and Methods

### 2.1. Chemicals and Biological Materials

The Anti-Boscalid monoclonal antibody [28] was obtained from the Institute of Agrochemistry and Food Technology, Spanish National Research Council (IATA-CSIC) located in Valencia, Spain. Monkey African green kidney (Vero) and Hamster adult kidney (HaK) cell cultures were originally sourced from LGC Promochem (Teddington, UK). Essential culture media components, Dulbecco’s Modified Eagle’s Medium (DMEM) basal medium, fetal bovine serum, horse serum, L-glutamine, antibiotics mixture (penicillin/streptomycin), and trypsin-EDTA solution were supplied by Biowest (Nuaillé, France). The QuEChERS extraction kit was acquired from Agilent Technologies (Lake Forest, CA, USA). Screen-printed electrodes for electrochemical measurements were obtained from Embio Diagnostics Ltd. (Nicosia, Cyprus). Boscalid, mandipropamid, and phosphate-buffered saline (PBS) were provided by Sigma-Aldrich (Taufkirchen, Germany).

### 2.2. Biosensor Manufacturing 

Both cell lines were cultured in DMEM basal medium alongside 10% FBS, 1% antibiotics mixture, and 1 mM L-glutamine in a chamber (5% CO_2_, 37 °C). The adherent cells were dissociated from the culture flask, after incubation with trypsin-EDTA for a period of approximately 5 min in the culture chamber. Then, a centrifugation step (2 min at 1200 rpm) was performed to concentrate the cell suspension. Anti-boscalid antibodies were utilized for cell membrane modification via electroinsertion. Specifically, a batch of cells, at a density of 2.5 × 10^6^ in 40 μL of phosphate-buffered saline, was incubated with 400 μL of antibody for 20 min on ice. Afterwards, the mixture was exposed to two square electric pulses, each one corresponding to an electric field of 1800 V/cm [41,43]. The membrane-engineered cells were placed overnight in the culture chamber. The next day, the cells were dissociated, concentrated, and tested against standard boscalid or mandipropamid solutions, as well as spiked lettuce samples. Different batches of cells were engineered with elevating concentrations of the boscalid antibody (0.5 μg/mL, 1 μg/mL, 2 μg/mL, and 5 μg/mL).

### 2.3. Point-of-Test Setup 

**Figure 1:**
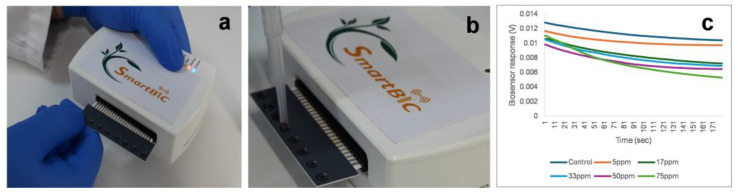
(**a**) The portable potentiometric biosensor device with the disposable sensor strip. (**b**) Membrane-engineered cells and sample were placed on the top of each of the eight carbon screen-printed electrodes for simultaneous measurements. (**c**) Testing results are displayed in real time on screen, as a voltage vs. time graph, and stored for later processing.

A portable multichannel potentiometer, measuring open circuit potential, customized by Embio Diagnostics Ltd., was used to record changes in engineered cell’s membrane potential and other electrical properties of the cells. This device connects via Bluetooth to a tablet device and measures the real-time electric property changes due to the presence of analytes binding to or being taken up by the membrane-engineered cells with boscalid antibodies. The potentiometer can perform up to eight simultaneous measurements using a disposable strip (Figure 1a) of eight carbon screen-printed electrodes with a carbon working electrode and an Ag/AgCl (silver/silver chloride) reference electrode. The system generates rapid, sensitive, and reproducible responses.

### 2.4. Standard Solutions Prepation and Measurement Process

Various standard solutions were prepared according to existing boscalid’s maximum residue limits (MRLs) for lettuce, i.e., the highest amount of pesticide residue legally allowed. The MRL was set at 50 ppm., according to Commission Regulation (EU) 2022/1324 [45], and concentrations above and below this specific level were prepared, at 5 ppm, 17 ppm, 33 ppm, 50 ppm, and 75 ppm. Measurements were taken by placing 45 μL of membrane-engineered cells (50 × 10^3^) on top of each electrode on the strip, and, immediately after, 5 μL of the sample was added (Figure 1b) to start the measurement. The cells’ responses to various samples (control and positive samples) were recorded as a time series of potentiometric measurements (V). Each sample was measured for 180 s, with data recorded at a sampling rate of 2 Hz (Figure 1c). The experiments have been performed by the same device according to manufacturer’s instructions for setup and maintenance.

### 2.5. Spiked Lettuce Samples—Sample Preparation

Samples were prepared according to solid phase extraction method, as is recommended by EU authorities (European Method EN 15662) [46]. QuEChERS extraction was carried out, employing the Bond Elut QuEChERS P/N 5982 extraction kit consisting of 4 g of MgSO_4_ (magnesium sulfate) 1 g of NaCl (sodium chloride), 1 g of Na_3_C_6_H_5_O_7_ (sodium citrate), and 0.5 g of C_12_H_18_Na_4_O_17_ (disodium citrate sesquihydrate). Pigments were removed by the dispersive solid-phase extraction kit, Bond Elut QuEChERS P/N 5982-5221. To evaluate possible matrix effects, spiked lettuce samples was prepared with boscalid solutions of known concentration. Samples without boscalid were used as control. In a 50 mL centrifuge tube, a ceramic homogenizer and 2 g of spiked lettuce tissue were mixed with 10 mL of organic-free water, 10 mL of acetonitrile, and the QuEChERS extraction kit (P/N 5982) packet (EN 15662). The sample was homogenized manually with vigorous shaking for 1 min. The mixture was then centrifuged for 5 min at 3000× *g* to collect the top organic layer in 1 mL aliquots. Next, 1 mL of the acetonitrile phase was added to the second kit (QuEChERS dispersive kit P/N 5982-5221) for pigment removal. Then, 2 min of moderate stirring was performed, followed by centrifugation (5 min, 3000× *g*), and 0.5 mL of the supernatant was collected and dried by evaporation under a constant nitrogen stream [46,47]. The residues were recovered in water and concentrations were adjusted in phosphate-buffered saline before testing.

### 2.6. Experimental Design and Data Analysis

The present study was designed as follows. Firstly, two cell lines, HaK and Vero, were membrane-engineered with various concentrations of boscalid antibody (0.5, 1, 2, or 5 μg/mL) and tested as biorecognition elements. Vero cells and a relative low concentration (0.5 μg/mL) of the antibody were selected for sensor fabrication. Then, biosensor responses were tested against standard solutions of boscalid (5 ppm, 17 ppm, 33 ppm, 50 ppm, and 75 ppm), with concentrations standing below, equal to, and above MRL. The selectivity of the system was assessed by testing membrane-engineered Vero cells with no antibody against boscalid standard solutions, to ensure that the system generates responses only after the selective biorecognition of boscalid from electroinserted antibodies. Mandipropamid, a fungicide regularly applied in lettuce culture, was employed to assess sensor’s cross-reactivity. Mandipropamid was tested because the established legal limit for the pesticide in lettuce is set at a high concentration of 25 ppm [45]. Finally, spiked lettuce samples with concentrations equivalent to standard solutions were tested to assess matrix effects on the biosensor’s performance. 

Every measurement is a data series of 360 records (Volt) taken within 180 s. Each sample (control, standard solution, and spiked sample) was tested six times, and four technical replicates were measured in each set (*n* = 24). Averages of all measurements were calculated. The means of the control values were estimated and used for corresponding samples’ data normalization, as follows:

$$normalised\;response=\frac{average}{mean\;control\;value}$$

Each tested sample generated 24 values of normalized responses. The presented normalized biosensor responses are the mean ± SEM of normalized responses.

Statistical differences between groups were determined by one-way ANOVA performed with GraphPad Prism (GraphPad Software, version 6 for Windows, San Diego, CA, USA), with a significance level set at *p* < 0.05.

## 3. Results

### 3.1. Biosensor Response to the Presence of Standard Boscalid Solutions

**Figure 2:**
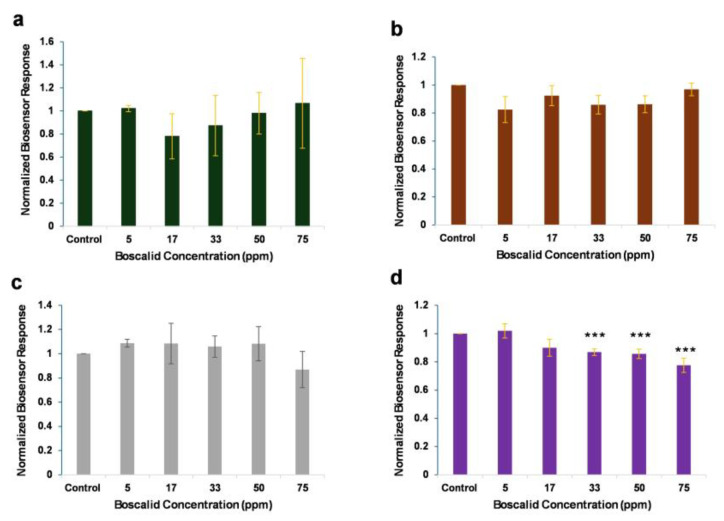
HaK-cell-based sensor responses to increasing boscalid concentrations. Sensor responses are presented after normalization to control (no treatment) value (sample/control). Cells are membrane-engineered with increasing concentrations of anti-boscalid antibody: (**a**) 0.5 μg/mL, (**b**) 1 μg/mL, (**c**) 2 μg/mL, and (**d**) 5 μg/mL. Data are means ± SEM (*n* = 24), received from six independent experiments with different batches of cells. ***: statistically significantly different results from the control (*p* < 0.001).

Two cell lines were employed as biorecognition elements, and batches of these cells were engineered with increasing concentrations of anti-boscalid antibody, at 0.5–5 μg/mL. Then, the cells were subjected to various concentrations of boscalid standard solutions, to identify the most susceptive cell line and the optimum antibody concentration. The responses of membrane-engineered HaΚ cells, with various concentrations of anti-boscalid antibody, against increasing concentrations of boscalid are presented in Figure 2.

The normalized biosensor responses indicate that membrane-engineered HaK cells with anti-boscalid antibody, in concentrations lower than 5 μg/mL, did not generate efficient responses for pesticide detection. Cells incubated with a high antibody concentration (5 μg/mL) demonstrated concentration-dependent responses, as the measurements were significantly different from the control at boscalid concentrations higher than 33 ppm. Following the operational principle of the integrated BERA/membrane-engineering method, the interaction between a target analyte (boscalid) and cells modified with a specific antibody for the analyte induces a variation in the cell membrane potential, which correlates with the concentration of the analyte.

**Figure 3:**
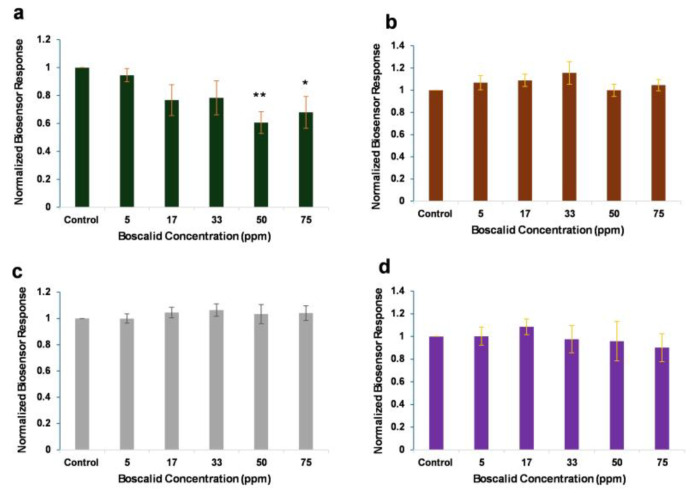
Vero-cell-based sensor responses to increasing boscalid concentrations. Sensor responses are presented after normalization to control (no treatment) value (sample/control). Cells are membrane-engineered with increasing concentrations of anti-boscalid antibody: (**a**) 0.5 μg/mL, (**b**) 1 μg/mL, (**c**) 2 μg/mL, and (**d**) 5 μg/mL. Data are means ± SEM (*n* = 24), received from six independent experiments with different batches of cells. *: significantly different from the control (*p* < 0.05), **: significantly different from the control (*p* < 0.01).

The responses of membrane-engineered Vero cells, with various concentrations of anti-boscalid antibody, against increasing concentrations of boscalid are presented in Figure 3. Membrane-engineered Vero cells with 1–5 μg/mL of anti-boscalid antibody did not produce a significantly different response to different, increasing boscalid concentrations. On the contrary, by decreasing the electroinserted anti-boscalid antibody concentration to 0.5 μg/mL, the observed pattern of the concentration-dependent decrease in cell membrane potential was more significant. The fact that the lower anti-boscalid antibody concentration (0.5 μg/mL) produced the best results can be explained as follows: consistent with prior findings [44], elevating the density of electroinserted antibodies on membrane-engineered cells and/or surpassing a certain threshold in the target analyte concentration does not result in a titrimetric interaction between the analyte (boscalid) and the membrane-engineered carrier cells containing the anti-boscalid antibodies.

The phenomenon arises from the interaction between the analyte and the electroinserted antibody, which induces electromechanical stress at the antibody site on the membrane. This stress prompts alterations in membrane properties such as conductivity and porosity [37]. Often, there is a constraint on modifying the cell membrane potential due to higher densities of electroinserted antibodies, wherein lower densities yield more a precise response resolution. The system presented distinct responses, after the addition of boscalid at concentrations above the 50 ppm (MRL) threshold. It is observed that, even though the responses derived after the 75 ppm treatment were statistically indifferent to those derived from the 50 ppm treatment, the larger concentration varies from the control value at a less significant probability level (*p*-value < 0.05). This can be attributed to the hook effect; that is, as the concentration of the analyte increases above a certain point, the system becomes saturated, and the signal begins to decline [38,48].

**Figure 4:**
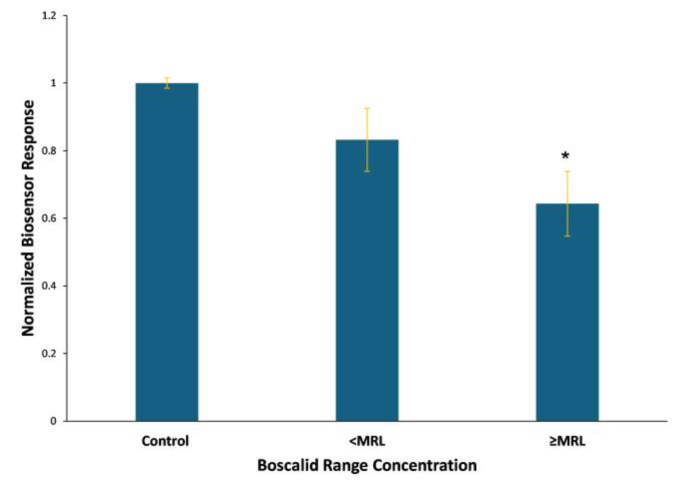
Cumulative Vero-cell-based sensor responses against boscalid range concentrations above and below MRL, which is 50 ppm for lettuce. Vero cells were membrane-engineered after the electroinsertion of 0.5 μg/mL of boscalid antibody. Sensor responses are presented after normalization to control (no treatment) value (sample/control). Data are means ± SEM (*n* = 24), received from six independent experiments with different batches of cells. *: significantly different from the control (*p* < 0.05).

The statistical analysis (*p* < 0.05) indicates that the Vero-cell-based sensor positively identified boscalid concentrations standing higher than the maximum residue level, whereas the HaK-cell-based biosensor generated indistinguishable signals at concentrations higher than 33 ppm. Cumulative Vero-cell-based biosensor responses against standard boscalid range concentrations, above and below the MRL, are presented in Figure 4. The system generates distinct responses against standard solutions with concentrations exceeding the existing maximum residue levels of the pesticide.

### 3.2. Proof of Biosensor Selectivity 

**Figure 5:**
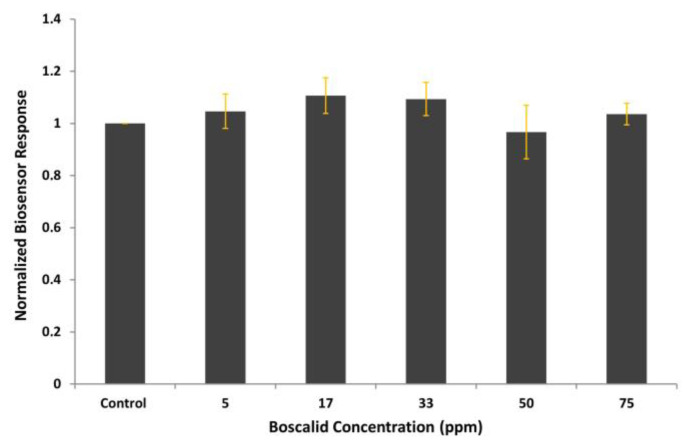
Vero-cell-based sensor responses to increasing boscalid concentrations. Cells were subjected to electroinsertion with no boscalid antibodies. Sensor responses are presented after normalization to control (no treatment) value (sample/control). Data are means ± SEM (*n* = 24), received from six independent experiments with different batches of cells.

Vero cells were subjected to electroinsertion, without the addition of antibodies during incubation, and tested against increasing concentrations of the pesticide. The sensor responses are presented in Figure 5. The uniformity of bioelectric signals validates that the biosensor generates responses only after the selective biorecognition of boscalid from electroinserted antibodies. 

**Figure 6:**
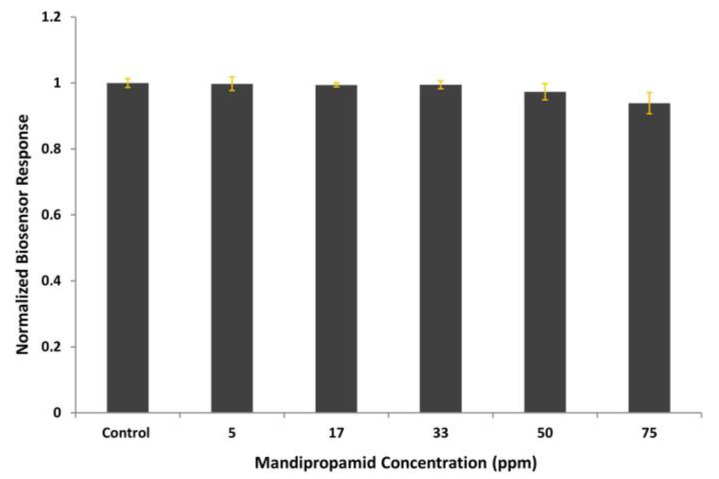
Vero-cell-based sensor response to different mandipropamid concentrations after elec-troinserting 0.5 μg/mL boscalid antibody in membrane-engineered cells. Sensor responses are presented after normalization to control (no treatment) value (sample/control). Data are means ± SEM (*n* = 24), received from six independent experiments with different batches of cells.

To elucidate possible interactions with non-target analytes, the Vero-cell-based sensor was tested against mandipropamid, a pesticide commonly used in lettuce culture [49]. Various concentrations of mandipropamid, proportional to the existing maximum residue levels [50], were applied, and biosensor responses are presented in Figure 6. Cross-reactivity is not observed. 

### 3.3. Biosensor Response to the Presence of Spiked Boscalid in Lettuce Leaf Extracts

**Figure 7:**
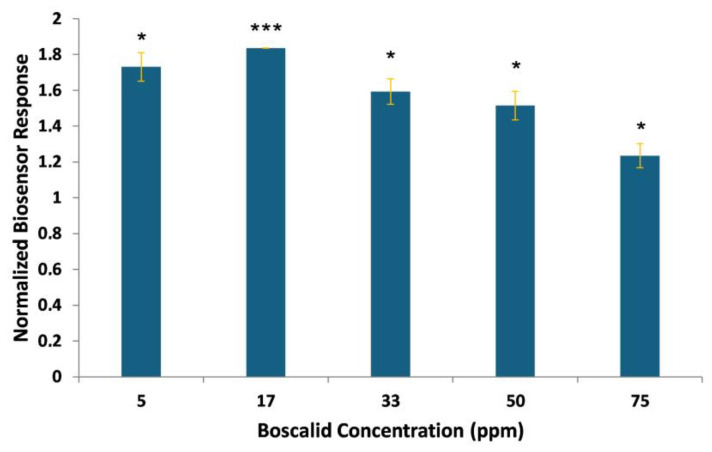
Vero-cell-based sensor responses against lettuce samples spiked with known boscalid concentrations. Vero cells were membrane-engineered after the electroinsertion of 0.5 μg/mL of boscalid antibody. Sensor responses are presented after normalization to control (no treatment) value (sample/control). Data are means ± SEM (*n* = 24), received from six independent experiments with different batches of cells. *: significantly different from the control (*p* < 0.05), ***: significantly different from the control (*p* < 0.001).

The Vero-cell-based biosensor’s feasibility for routine analysis was tested against spiked lettuce extracts for assessing the matrix effects on the biosensor’s performance. Specifically, lettuce tissues were sprayed with several boscalid concentrations, above, equal to, and below the existing legally tolerated limits, subjected to QuEChERS extraction, and analyzed. Biosensor responses against the spiked samples are presented in Figure 7. 

**Figure 8:**
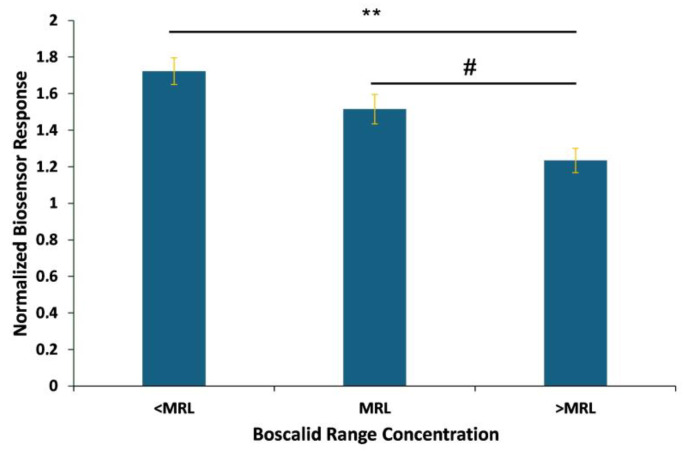
Cumulative Vero-cell-based sensor responses against lettuce samples spiked with known boscalid concentrations standing above, equal to, and below MRL, which is 50 ppm for lettuce. Vero cells were membrane-engineered after the electroinsertion of 0.5 μg/mL of boscalid antibody. Sensor responses are presented after normalization to control (no treatment) value (sample/control). Data are means ± SEM (*n* = 24), received from six independent experiments with different batches of cells. **: significantly different from ΜRL values, (*p* < 0.01), #: significantly different from MRL value (*p* < 0.05).

The samples are classified in three concentration-dependent groups above, equal to, and below the MRL, confirming the proper functioning of the system. The observed results (Figure 8) showed a significant response between different concentrations, distinguished according to the MRL. The sensor generated responses above control levels when applied against spiked samples obtained after the extraction and purification steps. In contrast, testing the standard solutions produced results lower than those of the control. An analogous pattern has been observed in another assay [39] and it is attributed to the matrix effect due to the extraction process. 

Cumulative responses, i.e., the average of the experimental replications, indicate that the biosensor was able to detect boscalid at different concentration ranges, making the system suitable for the detection of boscalid in lettuce samples, since it could detect levels below, equal to, and above the established legal MRL, according to Regulation (EC) 2022/1324 [45]. 

## 4. Discussion

Lettuce (*Lactuca sativa* L.) holds a distinguished status among leafy vegetables worldwide for its taste, nutritional profile, and antioxidant properties. A substantial portion of the global leafy vegetable market relies on lettuce production, and various pesticide monitoring techniques are utilized to ensure public health and environmental protection [51]. Conventional detection strategies such as chromatography and ELISA methods are highly effective and sensitive; however, they rely on laboratory-based, time-consuming techniques, so their on-site application in agricultural fields is largely restricted. Recent advances promote on-site sensing strategies and portable devices for detecting pesticide residues in agricultural foods [52]. 

Biosensors are a promising solution for the accurate, rapid, sensitive, and portable detection of pesticides in agricultural products. A biosensor typically utilizes biological recognition elements, such as enzymes, antibodies, and those which specifically bind to the analyte. Biosensors can employ various transduction principles, including electrochemical, optical, or piezoelectric methods, to detect and quantify pesticide residues. They provide rapid detection capabilities, often yielding results in minutes, compared to the hours required for a laboratory analysis. Moreover, biosensors can be miniaturized and designed for on-site or in-field testing, enabling real-time monitoring directly at the agricultural site or during processing and distribution [53,54]. Various types of biosensors have been developed for the detection of residues in lettuce, against a vast number of pesticides such as carbendazim [55], chlorpyrifos [56,57], paraquat [58], carbofuran [59], and malathion [60,61]. Moreover, a surface plasmon resonance (SPR)-based electrochemical immunosensor has been proposed for the detection of boscalid in lettuce. The system displayed sufficient sensitivity for detecting pesticide concentrations near the MRL [62]. 

The suggested biosensor is manufactured according to the standards of the Bioelectric Recognition Assay and MIME technology. This technology has been cited in the advancement of biosensors designed for detecting various analytes, including viruses, biomolecules, and pesticides. These systems have showcased notable benefits in terms of their rapid measurement execution and versatile application owing to their portability [44]. Particularly, the presented biosensor is constituted by a membrane-engineered Vero cell suspension, placed over the sensing unit, a disposable set of eight screen-printed carbon electrodes. When exposed to boscalid, cells undergo rapid and specific changes in their membrane potential, that is recorded by a specialized portable potentiometer, capable of monitoring real-time fluctuations of the electric characteristics of cells. The instrument can read up to eight concurrent measurements, in less than 3 min. The results are transmitted via Bluetooth technology to a smartphone/tablet device, equipped with a customized user interface, for remote monitoring and analysis. The portability of the system is ensured by employing the QuEChERS method, for the lettuce sample pretreatment. QuEChERS is a fast and feasible technique for pesticide detection, appropriate for on-the-spot applications [63].

Vero cells demonstrated significant biorecognition properties against boscalid, after the electroinsertion of antibodies, at a concentration of 0.5 μg/mL. Despite the significant sensitivity exhibited by the membrane-engineered HaK cells to low concentrations of the analyte, their responses reached a plateau and the MRL threshold was not detectable. Whole-cell-based biosensors are advantageous systems for the detection of diverse substances or analytes within intricate samples. These biosensors incorporate living cells onto a suitable substrate or platform, where they interact with the target analyte, providing distinct benefits like an exceptional specificity, adaptability, and responsiveness. In contrast to traditional biosensors, whole-cell-based biosensors exhibit the capability to identify a broader array of substances, rendering them more responsive to alterations in the electrochemical status of tissue samples, other cells, or the environment [64,65,66]. To present enhanced biorecognition abilities, the cells can be engineered to express specific biomolecules, such as receptors or enzymes, that recognize and respond to the target analyte. Moreover, the cells’ modification contributes to their function across a wider spectrum of conditions, encompassing diverse temperatures and pH levels [67,68]. MIME technology utilizes the incorporation of specific antibodies onto the cells’ membrane as recognition elements, that offer a high selectivity and affinity for specific molecules. In this case, membrane-engineered Vero cells presented enhanced biorecognition properties against the pesticide, while non-engineered Vero cells did not generate distinct signals. The electroinsertion of the antibodies was the critical process for the transformation of Vero cells into specific biorecognition elements. Moreover, the system was selective for boscalid identification since no cross-reactivity against mandipropamid was reported. In another study, Apostolou et al. [69] presented a portable biosensor system based on BERA and MIME principles, suitable for screening acetamiprid residues in lettuce. The system’s responses against lettuce samples were also ranked within 3 min as below or above the maximum residue levels. The rapid detection and classification of residue levels without necessitating the specialized processing of the measurements highlights the system’s capability for mass screening by non-experts.

Taken together, the system is a very promising screening tool for the on-site and rapid detection of boscalid residues in lettuce. The biosensor offers the advantage of direct monitoring, since it can process up to eight samples in 3 min, and it can be operated by non-specialized personnel. In advance, this ready-to-use platform is connected wirelessly to a smartphone/tablet device enabling remote data processing and the direct classification of samples according to the existing MRL.

## 5. Conclusions

Biosensors hold promise in complementing or replacing traditional analytical techniques for monitoring pesticides in agricultural produce, thereby ensuring food safety and environmental sustainability. These platforms offer fast, sensitive, and on-the-spot detection capabilities, eliminating the need to transport samples to distant laboratories, thus conserving time and resources. The ongoing advancement and practical deployment of the proposed biosensor system in this study could expedite the implementation of cutting-edge technologies in sustainable agricultural practices. The Vero-whole-cell-based sensor presents a promising approach due to its sensitivity, portability, and capability to conduct eight independent measurements within 3 min. Further experiments will explore the feasibility of the system for food safety diagnostics. Overall, continued research and development efforts will address the current limitations and enhance the utility of the biosensor in safeguarding human health and the environment from the adverse effects of pesticide exposure.

## Data Availability

The data presented in this study are available upon request.
